# New Orleans Medical Journal, Devoted to the Cultivation of Medicine and the Associate Sciences

**Published:** 1845-03

**Authors:** 


*New Orleans Medical Journal, devoted to the Cultivation of Medicine and the Associate Sciences.* Edited by ERASMUS D. FENNER, M. D., and A. HESTER, M. D.

WE had intended to have noticed the above named Journal in our last, but were prevented for want of room. It was commenced in June, 1844, and is published bi-monthly. Each number contains 128 pages, and is divided into four parts. The first is devoted to original communications; the second, embraces a periscope of practical medicine; the third, health of the city, with reports from the New Orleans Hospital, and the fourth, notices of recent medical publications. It is, in every respect, ably conducted.
[*Balt. Ed.*

